# MFCA-Transformer: Modulation Signal Recognition Based on Multidimensional Feature Fusion

**DOI:** 10.3390/s25165061

**Published:** 2025-08-14

**Authors:** Xiao Hu, Mingju Chen, Xingyue Zhang, Jie Rao, Senyuan Li, Xiaofei Song

**Affiliations:** 1 School of Automation and Information Engineering, Sichuan University of Science and Engineering, Yibin 644005, China; 323085404103@stu.suse.edu.cn (X.H.); 323081104117@stu.suse.edu.cn (X.Z.); 323085404110@stu.suse.edu.cn (J.R.); 323085404108@stu.suse.edu.cn (S.L.); 323085404418@stu.suse.edu.cn (X.S.); 2 Intelligent Perception and Control Key Laboratory of Sichuan Province, Sichuan University of Science and Engineering, Yibin 644005, China

**Keywords:** modulation recognition, feature extraction, multi-dimensional feature fusion, attention mechanism

## Abstract

In order to solve the problems of modulation signals in low signal-to-noise ratio (SNR), such as poor feature extraction ability, strong dependence on single modal data, and insufficient recognition accuracy, this paper proposes a multi-dimensional feature MFCA-transformer recognition network that integrates phase, frequency and power information. The network uses Triple Dynamic Feature Fusion (TDFF) to fuse constellation, time-frequency, and power spectrum features through the adaptive dynamic mechanism to improve the quality of feature fusion. A Channel Prior Convolutional Attention (CPCA) module is introduced to solve the problem of insufficient information interaction between different channels in multi-dimensional feature recognition tasks, promote information transmission between various feature channels, and enhance the recognition ability of the model for complex features. The label smoothing technique is added to the loss function to reduce the overfitting of the model to the specific label and improve the generalization ability of the model by adjusting the distribution of the real label. Experiments show that the recognition accuracy of the proposed method is significantly improved on the public datasets, at high signal-to-noise ratios, the recognition accuracy can reach 93.2%, which is 3% to 14% higher than those of the existing deep learning recognition methods.

## 1. Introduction

Sensor technology plays a vital role in modern communication systems; the signal captured by the sensor contains rich modulation information, and the modulation signal identification is the core of decoding the key information. Signals collected by sensors in wireless communication, radar or military communication are usually processed by various modulations, which affect the transmission efficiency, anti-interference ability, and information-carrying capacity of signals. Modulation signal recognition technology is the bridge between sensor perception and signal analysis. The acquisition accuracy, response speed, and anti-noise ability of the sensor affect the recognition accuracy. The recognition results can optimize the sensor acquisition strategy and improve the system efficiency. Especially in the complex electromagnetic environment, the receiving parameters can be dynamically adjusted to reduce invalid data. In these systems, signal analysis and processing is the basis of efficient communication, and modulation recognition is one of the important technologies, which automatically identifies the modulation type of the received signal through analysis [1], providing support for demodulation and data decoding. It is widely applied in fields such as wireless communication, radar, and military communication. Conventional approaches for modulation classification can be broadly categorized into two main groups: likelihood-based and feature-based techniques [2]. The likelihood-based method relies on prior knowledge, which is computationally complex and has low recognition accuracy in complex environments. Based on the feature rule, the high-order moment [3], cyclic spectra [4], cyclic statistics [5] and other features of the signal are extracted, and then the classifier is used for recognition. Although likelihood-based methods are computationally simple, they require a large number of empirical features and perform poorly in complex scenarios. This is particularly true in cases with few samples, where it is challenging to effectively extract appropriate features for recognition. Therefore, modulation recognition technology needs further optimization and innovation when facing complex signal environments.

Deep learning technology has achieved remarkable results in automatic feature extraction and classification. O’Shea et al. [6] introduced an automatic modulation recognition model based on CNN, which achieved higher recognition performance compared to traditional methods relying solely on manually extracted expert-crafted features. Peng et al. [7] employed a deep convolutional neural network for automatic modulation recognition. Liu et al. [8] proposed a DCN-BiLSTM model for AMR, which extracts the phase and amplitude characteristics of the modulated signal through DCN, and the BiLSTM layer solves the long-term correlation problem. Tao et al. [9] proposed the AG-CNN model, which integrates the dual attention mechanism and Ghost module to improve performance while reducing the number of parameters. Li et al. [10] proposed the LAGNet model, integrating LSTM and GCN, and used an attention mechanism to enhance signal recognition capability. Elsagheer et al. [11] combined ResNet with LSTM, improving classification accuracy under high signal-to-noise ratios (SNR). Zhang et al. [12] proposed a novel network called CTRNet, which combines CNN and Transformer to capture global sequential dependencies and optimize parameter efficiency. Chen et al. [13] enhanced training performance by generating augmented samples through wavelet transform. Asad [14] proposed an innovative method based on the linear combination of cumulants (LC) and genetic algorithm (GA), which uses hypercumulants to classify five modulation types, and uses a k-nearest neighbor classifier (KNN) to improve the recognition rate under low SNR, which outperforms the existing methods. Gao et al. [15] proposed a framework based on a Mixture of Experts model, where a Transformer module processes low-SNR signals and a ResNet module handles high SNR signals, achieving effective recognition under varying SNR conditions. SCTMR-Net, proposed by An et al. [16] achieves better performance by using constant wavelet convolution filters in the SCT module employed, reducing the learning parameters and lowering the prior channel state. The method based on MFDE proposed by Li [17] can effectively reflect the complexity, frequency, and amplitude changes in the signal in the feature extraction of the noise signal. Esmaiel et al. [18] proposed a feature extraction method combining Enhanced Variational Mode Decomposition, Weighted Permutation Entropy, and Local Tangent Space Alignment. The method first decomposes the signal into intrinsic mode functions, calculates the WPE of each IMF to enhance the decomposition effect of VMD, and then uses LTSA to reduce the high-dimensional features to two-dimensional. Zhang et al. [19] proposed to build a lightweight neural network, MobileViT, driven by clustering constellation images to achieve real-time automatic modulation recognition, which has superior performance and efficiency. Zhang et al. [20] proposed a real-time automatic modulation recognition method based on a lightweight mobile radio Transformer, which is constructed by iteratively training the radio Transformer and pruning the redundant weights with information entropy. It learns robust modulation knowledge from multi-modal signal representation and has strong adaptability to communication conditions. The method can be deployed on an unmanned aerial vehicle receiver to realize air-to-air and air-to-ground cognitive communication in low-requirement communication scenarios.

For certain modulation schemes, their performance in specific environments may be highly similar, making it difficult to distinguish them effectively using a single feature. Therefore, multi-feature fusion methods have become crucial for improving recognition accuracy. By integrating multiple feature types, models can more accurately identify modulation schemes under complex environments and noise interference. Liu et al. [21] proposed a novel AMC method for OTFS systems. We constructed a two-stream convolutional neural network model to capture multi-domain signal features simultaneously, which greatly improves the recognition accuracy. Hu et al. [22] proposed a KB-DBCN network, which combines knowledge-based feature extraction and data-driven methods to process signal features in parallel, and maps and fuse features through fully connected layers. Zhang et al. [23] proposed a recognition scheme based on multi-modal feature fusion, which constructs a deep convolutional neural network to extract spatial features through RSBU-CW and interacts with temporal features extracted by LSTM to enhance feature diversity. Hao et al. [24] proposed a modulation classification framework using multi-domain amplitude features, achieving recognition through statistical analysis of envelope variations. Zhao et al. [25] proposed a hybrid feature extraction method based on time-domain statistical features and high-order cumulants and introduced a new parameter, AT, to significantly improve the modulation recognition performance under low-SNR conditions. Gao et al. [26] normalized and fused time-frequency entropy features, higher-order statistical features, and network-extracted features using non-negative matrix factorization, achieving effective signal recognition. Tan et al. [27] proposed a multi-feature fusion approach using IQ signals, AP signals, and time-frequency signals to construct a more comprehensive signal representation, enhancing the feature expression capability and recognition accuracy of automatic modulation recognition methods. Wang et al. [28] proposed a multi-scale network with multi-channel input and multi-head self-attention and BiGRU, but it has the problems of a large number of parameters and high computational complexity. Liu et al. [29] proposed a multi-modal data fusion method based on iDCAM, which constructs feature maps utilizing local and global embedding layers, in which the inputs pass through iDCAM modules to capture high-level features and global weights, and modal advantages are combined to improve the recognition effect. Wei et al. [30] introduced a novel multi-dimensional shrinkage module based on CNN to effectively extract temporal information from raw IQ signals, though it still suffers from relatively high time and space complexity. Jiang et al. [31] proposed a BLR modulation recognition method, which combines BiLSTM to extract the timing features of IQ data, ResNet-18 to extract the constellation features, and serial feature fusion to mine the complementarity of multi-modal data.

Although existing research has made progress in improving recognition performance under noisy environments, many challenges remain in complex situations. In particular, under high noise interference or poor channel conditions, the performance of modulation recognition models may significantly decrease. To address this critical issue, the Multi-Feature Fusion Channel Attention Transformer (MFCA-Transformer) proposed in this paper achieves performance breakthroughs through the following innovative work:

- By extracting multi-dimensional information from different features in the original data set, including constellation features, time-frequency features, and power spectrum features, the construction of multi-dimensional feature maps not only provides a more comprehensive representation of the data, fully exploiting its potential features, but also helps the model better recognize and classify complex patterns, achieving higher accuracy and effectiveness.
- Traditional feature fusion methods, such as direct concatenation or simple convolution, are unable to fully utilize global information, resulting in fused features that are not comprehensive and precise. In this paper, a Triple Dynamic Feature Fusion (TDFF) is proposed. By introducing a dynamic mechanism, it adaptively fuses local features at different scales based on global information, improving the quality of feature fusion and enhancing the model’s recognition ability in complex environments.
- For the issue in Swin Transformer models where there is insufficient information interaction between feature channels in multi-dimensional feature recognition tasks, this paper introduces the Channel Prior Convolutional Attention (CPCA) mechanism, which effectively promotes interaction and information transfer between feature channels, compensating for the deficiencies of traditional models in channel information interaction. As a result, the model’s accuracy and robustness in handling multi-dimensional feature classification tasks are improved.
- When dealing with modulation signals under low SNR, label noise may exist, causing the model to overfit the training data, and leading to poor generalization on low-SNR signals. Therefore, label smoothing is introduced into the standard cross-entropy loss function. By adjusting the distribution of the true labels, the smoothed model’s predicted probabilities are made closer to the real confidence, thus enhancing the model’s generalization ability.

## 2. Methods

### 2.1. The MFCA-Transformer Recognition Network with Multi-Dimensional Feature Fusion

**Figure 1:**
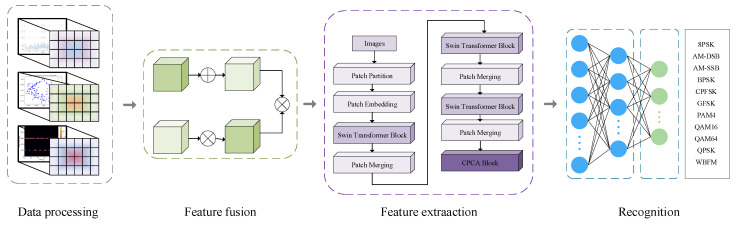
MFCA-Transformer structure diagram.

Based on the aforementioned research, this paper proposes a deep neural network architecture based on multi-dimensional feature input fusion, aiming to effectively improve the accuracy of signal processing and feature extraction. The network architecture primarily consists of four key modules: multi-dimensional feature construction, multi-dimensional feature fusion module, feature extraction module, and attention mechanism module. The structure of the MFCA-Transformer network model is shown in Figure 1.

After data preprocessing, multi-dimensional feature representation is constructed, including the constellation diagram feature of the modulation signal, the time-frequency diagram feature based on short-time Fourier transform, and the power spectrum diagram feature as the network input. The pre-processed features first enter the TDFF module, which integrates the features of different dimensions adaptively through the dynamic weight allocation mechanism, and outputs the fused feature vector with the same dimension. The fused features are fed into a neural network based on Swin Transformer architecture, which uses a hierarchical self-attention mechanism to model long-distance dependencies in the global range, and effectively captures local and global features in the image or signal through sliding window technology.

In the final stage of feature extraction, the output layer is replaced by the CPCA module. The CPCA module extracts the prior information of the channel dimension through the convolution operation, and optimizes the feature channel by combining with the squeeze-and-excitation mechanism to strengthen the key features and suppress noise interference, so as to further improve the feature discrimination. Finally, the optimized features are mapped to the classification space through the fully connected layer, and then the probability distribution of each class is calculated through the softmax layer to generate the final classification result.

### 2.2. Modulated Signal Multi-Dimensional Feature Construction

Modulated signals are primarily categorized into two types: analog modulation and digital modulation. Analog modulation directly encodes the original information onto a carrier signal, whereas digital modulation first converts digital signals into analog form before modulation or directly modulates the digital signals. In wireless communication systems, the transmitter is responsible for converting information into either analog or digital modulated signals for transmission. The channel serves as the medium for information transfer, and the receiver detects and decodes the signals. The received signal can be expressed as follows:

(1) $$r(t)=\int_{-\infty }^{\infty }s(\tau )\cdot h(t-\tau )d\tau +n(t)$$

where $s\left(\tau \right)$ denotes the transmitted modulated signal, $h\left(t\right)$ indicates the channel impulse response, and $n\left(t\right)$ corresponds to the additive noise signal. In this study, additive noise is employed to investigate modulation algorithms under complex channel conditions.

#### 2.2.1. Constellation Characteristics

Constellation feature representation is the core visualization tool used to describe the characteristics of modulation signals, and its essence is the set of projections of signal vectors on the complex plane. By representing different symbols or signal states on the complex plane, it helps us to intuitively understand the signal and its characteristics. For a discrete-time signal $s\left(t\right)=I\left(t\right)+jQ\left(t\right)$, the constellation points are located at coordinates $\left(E\left[I\right],E\left[Q\right]\right)$, where *I* (in-phase) and *Q* (quadrature) denote the orthogonal components. This can be represented mathematically as

(2) $$C=\left\{c_{k}=\right.\left.A_{k}e^{j\phi k}\right|\left.k=1,2,\ldots ,M\right\}$$

**Figure 2:**
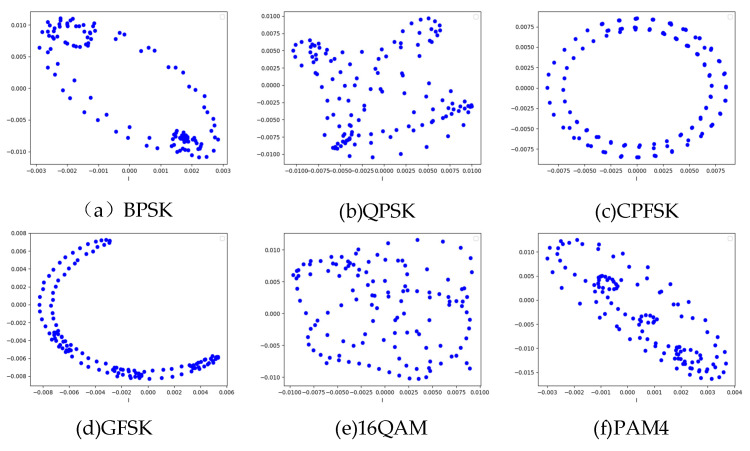
Constellation characteristics of six modulation schemes.

Here, $A_{k}$ and $\phi_{k}$ correspond to the discrete amplitude and phase values, respectively. *M* represents the modulation order, that is, the number of symbol states in a specific modulation scheme; for example, *M* = 16 for 16QAM modulation, *M* = 2 for BPSK modulation, which is used to quantify the symbol space complexity of different modulation signals. BPSK has two constellation points, which are located in the direction of the real axis on the complex plane and represent 1 bit of information. The phases of the constellation points are 0° and 180°, respectively. The 16QAM has 16 constellation points, each of which represents 4 bits of information, combining different amplitude and phase variations to achieve higher transmission rates. PAM4 uses 4 different amplitude levels to represent 2 bits of information and the points in the constellation are distributed on one dimension of the complex plane according to the amplitude values. The constellation feature map uses an oversampling mechanism to generate in-symbol samples, and the specific sampling rate is set to be 8 times of the symbol rate, that is, each symbol contains 8 sample points. The characteristic constellation diagrams for BPSK, QPSK, CPFSK, GFSK, 16QAM, and PAM4 modulation schemes at high SNR are shown in Figure 2.

#### 2.2.2. Time-Frequency Characteristics

**Figure 3:**
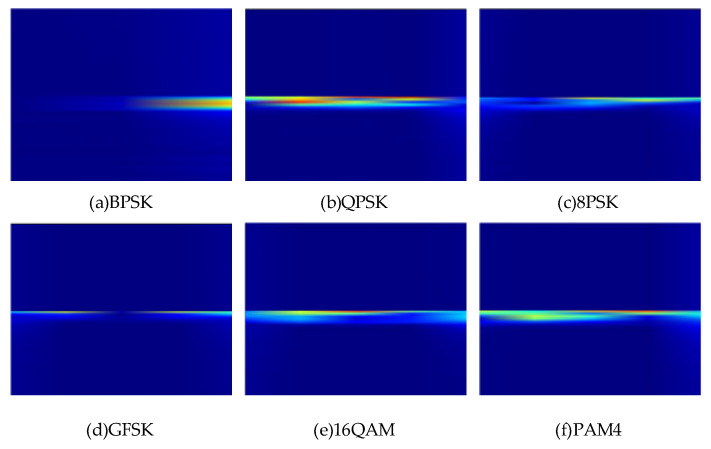
Time-frequency characteristics of six kinds of modulated signals.

With the continuous advancement of communication technologies, signal modulation schemes have become increasingly complex, rendering traditional time-domain or frequency-domain analysis methods progressively inadequate for effectively identifying these sophisticated modulated signals. Consequently, time-frequency analysis has emerged as a powerful tool for modulation recognition. Short-time Fourier transform (STFT), as a common time-frequency analysis method, divides the continuous time domain signal into several short-time segments by sliding window function. Fourier transform is applied to each segment to obtain the time-frequency domain distribution features. STFT is widely used in time-frequency representation and modulation recognition of signals, which provides a stronger ability to characterize the time-domain features of modulated signals. The mathematical formulation of the STFT is expressed as

(3) $$STFT\left\{\left.x(t)\right\}(t,f)\right.=\int_{-\infty }^{\infty }x(\tau )\cdot w(\tau -t)\cdot e^{-j2\pi f\tau }d\tau$$

where $x\left(t\right)$ is the signal to be analyzed, $w\left(t\right)$ is a window function, usually a windowing function, used to limit the time range of the signal, f is the frequency, τ is the time variable, and ∫ denotes the integration operation. The time-frequency characteristics of BPSK, QPSK, 8PSK, GFSK, 16QAM, and PAM4 modulation signals at high SNR are shown in Figure 3.

The time-frequency distribution of the signal is extracted by STFT. The horizontal axis implies the time series, the vertical axis corresponds to the frequency axis, and the color brightness reflects the energy density of the signal. The core differences in the six types of modulation signals are reflected in the form of energy distribution and time-frequency concentration: BPSK has a narrow band and linear distribution in the time-frequency plane because of its simple binary phase jump and fewer frequency components, and its energy is concentrated in a single frequency band; with the increase in modulation order, the phase jump of 8PSK becomes more intensive, the time-frequency energy band is further broadened, and the edge of time-frequency energy is slightly diffused; the time-frequency energy of GFSK presents a smooth band distribution, and there is no obvious discrete energy peak. The time-frequency energy band of 16QAM is the widest and dispersive, and the energy density decays rapidly with the frequency axis, reflecting the multi-dimensional characteristics of high-order modulation. The time-frequency energy of PAM4 is concentrated in the low-frequency band, and the amplitude modulation is dominated by the baseband energy.

#### 2.2.3. Power Spectrum Characteristics

**Figure 4:**
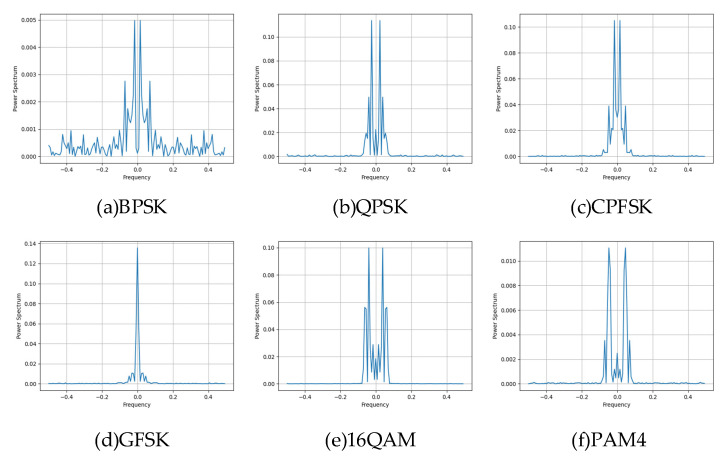
Power spectrum characteristics of six modulation signals.

The power spectral density of a modulated signal shows the signal’s power distribution across frequencies, reflecting energy variations. It helps evaluate bandwidth efficiency, spectral efficiency, and signal interference with noise. The power spectrum is defined as

(4) $$S(f)=\underset{T\to \infty }{\lim}\frac{1}{T}{\left|\int_{-T/2}^{T/2}x(t)e^{-j2\pi ft}dt\right|}^{2}$$

where $S\left(f\right)$ is the power spectrum of the signal $x\left(t\right)$, $\left|\cdot \right|$ represents the magnitude operation, and T is the observation time of the signal. Different modulation schemes have different power spectrum characteristics, which are determined by the symbol rate, bandwidth, and the modulation scheme itself. The power spectra of BPSK-, QPSK-, CPFSK-, GFSK-, 16QAM-, and PAM4-modulated signals at high SNR are shown in Figure 4.

The horizontal axis is the normalized frequency, and the vertical axis is the power spectrum density, which reflects the signal frequency domain energy distribution and out-of-band radiation. The core differences in the six types of modulation signals are mainlobe width, sidelobe suppression, and spectral efficiency: the mainlobe width of BPSK is the same as that of QPSK, and the sidelobes are high and dense; the sidelobes of QPSK are slightly lower than those of BPSK; the mainlobe of CPFSK is narrower than that of BPSK and QPSK, and the sidelobe suppression is better, which reflects the spectral advantages of constant envelope modulation. GFSK has very low sidelobes due to Gaussian filtering that suppresses out-of-band radiation, which is typical of spectrum-friendly modulation. The side lobe of 16QAM attenuates rapidly, and the energy is concentrated in the main lobe, which reflects the high spectral efficiency. PAM4 has the narrowest main lobe and almost leak-free side lobes, in contrast to the broadband nature of the other modulations.

### 2.3. TDFF Multidimensional Feature Fusion Module

**Figure 5:**
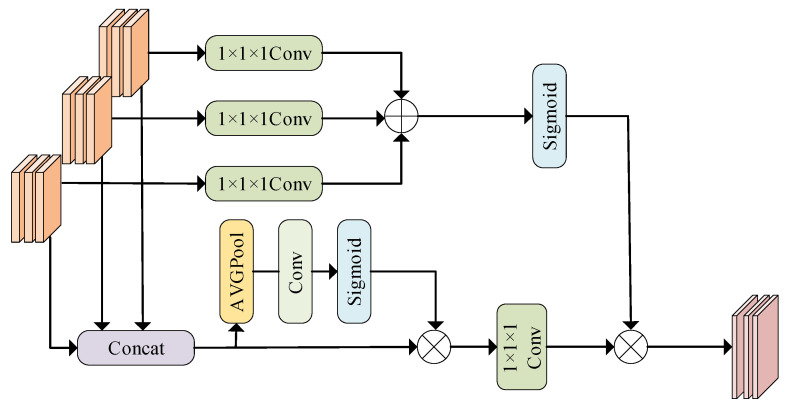
TDFF feature fusion module.

The existing feature fusion methods lack the use of global information, resulting in imperfect fusion features. In this paper, a three-input dynamic feature fusion (TDFF) module is proposed to optimize the effect of feature fusion through the dynamic adaptive fusion of multi-scale local features [32]. The dynamic mechanism includes channel dynamic selection and spatial dynamic selection. The former selects the important feature maps according to the global channel information, and the latter calibrates the feature maps according to the global spatial information, which enhances the detail preservation and global information utilization, enhancing the model’s segmentation accuracy and robustness. The TDFF module realizes the adaptive integration of local features through a global context-aware mechanism, as shown in Figure 5.

The TDFF module employs an adaptive feature selection mechanism guided by global contextual cues to optimize feature integration. First, the feature maps $F_{1}\in R^{C\times H\times W\times D}$, $F_{2}\in R^{C\times H\times W\times D}$, and $F_{3}\in R^{C\times H\times W\times D}$ are spliced into features $F\in R^{3C\times H\times W\times D}$ along the channel as follows:

(5) $$F=Concat\left(\left[F_{1};F_{2};F_{3}\right]\right)$$

In order to ensure that the subsequent modules can make full use of the fusion features, a channel compression mechanism needs to be introduced to recover the original channel dimensionality; the channel compression in TDFF does not simply use 1 × 1 × 1 convolution operation, but uses global channel information to guide the weight parameters $W_{C}$. The concatenated AVGPool is used to pool the stitching features globally, compress the spatial information, and focus on the channel-level global features. The Conv1 compresses the number of channels through 1 × 1 convolution, and then generates the channel attention weight through Sigmoid activation. This weight reflects the contribution of different dimensions and channels to the final fusion, and the larger the value is, the more important the corresponding channel is, so as to describe the weight distribution of features more effectively.

(6) $$W_{C}=Sigmoid\left(Conv_{1}\left(AVGPool\left(F\right)\right)\right)$$

where *W_c_* denotes the channel weight parameter.

The fused features are corrected through global channel information, the channel attention weight *W_c_* is multiplied by the original splicing feature *F* according to elements, and then 1 × 1 × 1 convolution channel dimension transformation is used to unify the multi-dimensional features to the same number of channels, so as to prepare dimensions for subsequent fusion. The convolutional layer adaptively enhances critical feature $F_{C}\in R^{X\times H\times W\times D}$ and filters out non-essential components under the guidance of channel-wise statistics,

(7) $$F_{C}=Conv_{1}\left(W_{C}⊗F\right)$$

where *F_c_* denotes the key feature map of the channel and ⊗ denotes the element-by-element multiplication.

In order to model the spatial dependence between local feature maps, The input feature maps *F*_1_, *F*_2_, and *F*_3_ are subjected to spatial information encoding using a 1 × 1 × 1 convolution, respectively. The convolution does not change the spatial dimension of the features, but fuses the local features in the channel, and the output dimension is consistent with the original feature map. The encoded multi-dimensional feature map is added element by element. This operation forces the multi-dimensional features to interact in the spatial dimension, and excavates the spatial association between features of different dimensions. Sigmoid activation is performed on the added feature map, and the values of all spatial positions are mapped to the [0, 1] interval to generate the global spatial attention weight *W_s_*.

(8) $$W_{S}=Sigmoid\left(Conv_{1}\left(F_{1}\right)⊕Conv_{1}\left(F_{2}\right)⊕Conv_{1}\left(F_{3}\right)\right)$$

where *W_s_* denotes global spatial information and ⊕ denotes element-by-element addition.

Multiplying the global spatial attention weight *W_s_* and the feature map *F_c_* after channel weighting pixel by pixel to obtain the final fusion feature $\hat{F}$. This spatial information is used to calibrate the feature map, highlighting salient spatial regions and thereby reinforcing the expression of key features.

(9) $$\hat{F}=W_{S}⊗F_{C}$$

In order to solve the problem of scale difference in multi-dimensional features, the two-step method of scale alignment is adopted, firstly, the constellation map features, time-frequency features and power spectrum features are mapped to 64 channels by 1 × 1 convolution to unify the channel dimensions, and then the features are unified to 64 × 64 spatial dimensions by adaptive average pooling, which makes the feature dimensions consistent and provides the basis for dynamic fusion. Different from the traditional fusion method of direct splicing or fixed weighting, TDFF realizes the dynamic adaptive fusion of multi-dimensional features through channel and spatial attention mechanism, so that the network can independently judge the features of different dimensions, channels, and spatial locations according to the characteristics of input signals.

### 2.4. Feature Extraction Module

**Figure 6:**
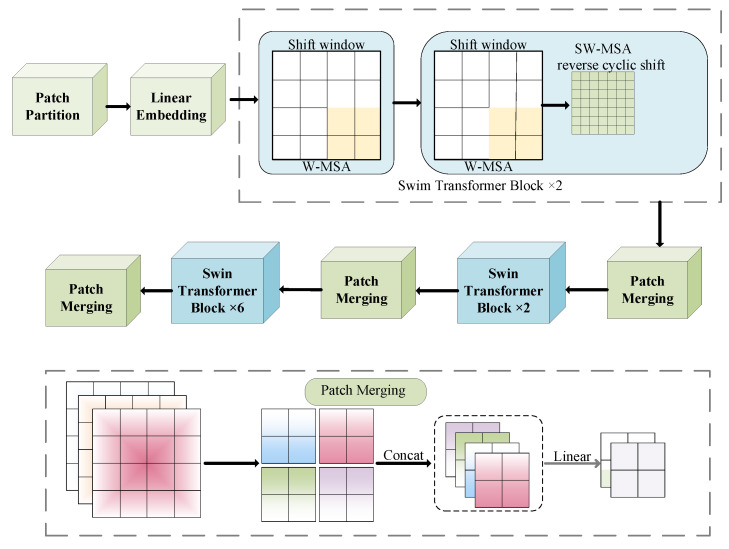
Swin Transformer network structure diagram.

The feature extraction part adopts the network architecture of Swin Transformer, which aims to improve the performance in computer vision tasks [33]. At present, various neural networks are used for image recognition. Compared with the recognition of modulation signals by various neural networks, the Swin Transformer neural network has strong scalability. It is suitable for the improvement of different network structures, and it has the best accuracy and speed trade-off. The structure of Swin Transformer is usually composed of several stages, and each stage contains several basic modules, namely, the Swin Transformer block [34]. The Swin Transformer network structure is shown in Figure 6.

In the Swin Transformer model, the input image is first divided into small blocks, and then the hierarchical structure progressively extracts features from the image data set. The input feature map (size: *H × W × C*) is partitioned into *N = (W/P) × (H/P)* non-overlapping patches, each of size *P × P*. Each small block is mapped to D dimension through linear mapping transformation, and the obtained image feature can be expressed by a formula, such as the following:

(10) $$Z_{0}=patchEmbedding(X)\in R^{N\times D}$$

where *Z*_0_ is the initial feature representation and *D* represents the embedding dimension. The layering process in the fusion Swin model is usually divided into multiple stages, and each stage contains multiple Swin Transformer blocks. The input feature map *Z*_0_ passes through L times of the Swin Transformer block, the feature dimension is unchanged, and the resulting feature map is denoted as *Z*_1_. The process is expressed by the following formula:

(11) $$Z_{1}=Swim\;Block\left(Z_{0}\right)(\mathrm{Repeat}\;L\;\mathrm{times})$$

A feature map Z_1_ is obtained, the resolution (spatial dimension) of which is reduced by half and the space size is changed from H × W to H/2 × W/2, and the number of channels is increased to 2D, so that the model can capture a wider range of context information. The feature maps are merged by the image block merging operation, which can be expressed by the following formula:

(12) $$Z_{2}=Patch\;Merging\left(Z_{1}\right)$$

After patch merging, adjacent blocks in *Z*_1_ are merged. This step strengthens the long-distance dependence, which is crucial to understand the global context, further aggregates the features of adjacent blocks, and enlarges the receptive field. The range of the feature map *Z*_2_ can be expressed as

(13) $$Z_{2}\in R^{\frac{N}{2}\times 2D}$$

After that, the model repeats this process, with the feature map going through several Swin Transformer blocks at each stage, and the patch merging operation is performed at the beginning of each stage. After this process is repeated many times, the feature map undergoes progressive downsampling while expanding its channel depth. The Swin Transformer block structure contains important operational processes in the model. Sliding the window attention, apply the self-attention mechanism to each window, and its expression is as follows:

(14) $$Z_{window}=Self\_Attention\left(Z_{window}\right)$$

After the window self-attention is completed, the two-layer feedforward network will deal with it. The FFN usually uses the GELU activation function and the linear layer to enhance the feature representation through nonlinear transformation. The function of the feed-forward network is to introduce a nonlinear transformation to further optimize feature after self-attention, and its expression is as follows:

(15) $$Z_{f’f’n}=FFN\left(Z_{\mathrm{window}}\right)$$

This leads to residual connection and layer normalization in the neural network. After each sub-module, the residual and normalization mechanisms ensure the stability of the deep Swin Transformer training and accelerate the model convergence. The function of residual connection and layer normalization is to stabilize the training process, optimize the gradient flow, and avoid the gradient disappearance or explosion during model training. The expression of residual connection and normalization operation is

(16) $$Z’=LayerNorm\left(Z+Z_{window}\right)$$

### 2.5. CPCA Module

Although the Swin Transformer model has powerful feature expression ability, it still has the issue of inadequate information interaction. CPCA [35] addresses limited inter-channel feature interaction in multi-dimensional recognition. By employing CPCA, the model strengthens attention on crucial image channel features, thereby improving classification accuracy.

**Figure 7:**
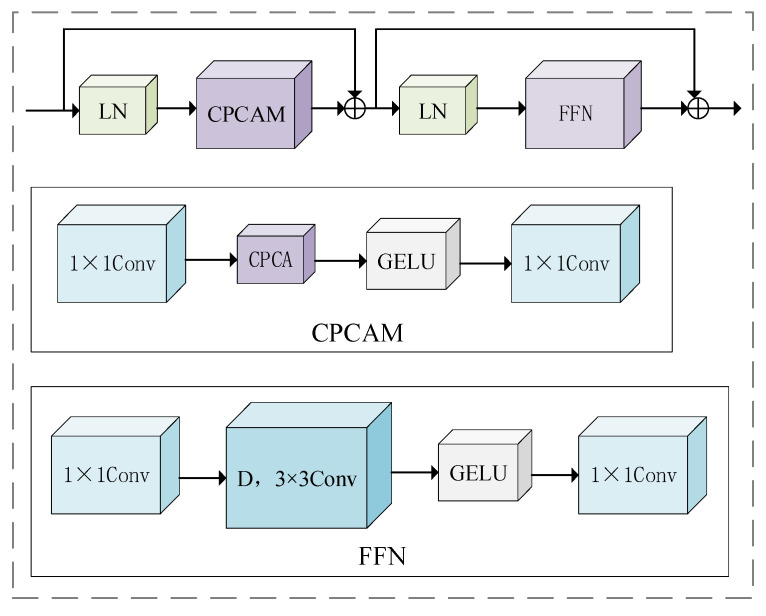
CPCA module structure.

The Swin Transformer block in the last stage of the Swin Transformer is replaced by a CPCA mechanism module, which captures the interaction between channels. It can deeply learn the dependence relationship between channel features, so as to guide the model, which is enabled to place greater emphasis on the feature channels containing key information. Unlike the traditional convolution operation that only focuses on local pixel information, the CPCA module designs special convolution kernels, which focus on processing the dependencies between channels, so that the model can adaptively adjust the learning of convolution kernels according to the interaction between feature channels. The CPCA module structure is shown in Figure 7.

**Figure 8:**
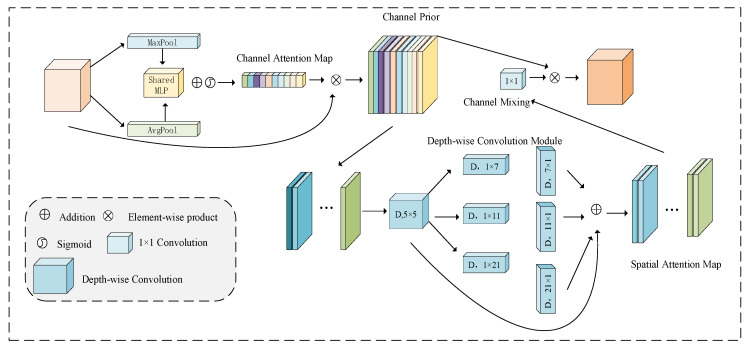
CPCA mechanism.

The CPCA module adopts an effective channel prior convolution attention, namely the CPCA mechanism, and supports the assignment of weights across both the channel and spatial dimensions. This is achieved by using multi-scale depth-separable convolutions and 1 × 1 bar convolutions, which efficiently extract spatial relationships while preserving channel priors. The multi-scale depth-wise strip convolution kernel enables efficient feature extraction with reduced computational cost. The CPCA mechanism integrates two key components, channel attention (CA) and spatial attention (SA), as shown in Figure 8.

The CA map is produced by the channel attention module, which captures the inter-channel relationships within the features. Following the CBAM method, spatial information in the feature map is gathered using average pooling and max-pooling, resulting in two independent spatial context features. These features are then fed into a shared multi-layer perceptron (MLP). The outputs from the shared MLP are combined through element-wise summation to form the final channel attention map. To reduce parameter overhead, the shared MLP is composed of a single hidden layer, with the activation size of the hidden layer being RCr×1×1, where r represents the reduction ratio.

(17) $$CA\left(F\right)=\delta \left(MLP\left(AvgPool\left(F\right)\right)+MLP\left(MaxPool\left(F\right)\right)\right)$$

where δ is the Sigmoid function.

The SA map captures cross-dimensional relationships, enabling dynamic weight allocation across both channel and spatial axes, rather than forcing consistency. Depth-wise convolution captures spatial relationships between features, preserving channel relationships while minimizing computational complexity. Additionally, a multi-scale structure is used to improve the convolutional ability to capture spatial relations. Finally, a convolution is applied at the end of the SA module for channel mixing, producing a more refined attention map.

(18) $$SA\left(F\right)=Conv_{1\times 1}\left(\sum_{i=0}^{3}Branch_{i}\left(DwConv\left(F\right)\right)\right)$$

where *DwConv* is the deep convolution, $Branch_{i},i\in \left\{0,1,2,3\right\}$ represents the *i*th branch, and $Branch_{0}$ represents the identity connection.

### 2.6. Loss Function

For most of the classification recognition tasks, the standard cross-entropy loss is adopted, that is, the real label is assumed to be one-hot encoding y (that is, *y_i_* = 1 indicates the real class, and the rest *y_j_* = 0). The unnormalized fraction (logits) of the model output is *z*, and the probability distribution *p* is obtained by softmax as follows:

(19) $$p_{i}=\frac{e^{zi}}{\sum_{j=1}^{C}e^{zj}}$$

The standard cross-entropy loss is

(20) $$L_{CE}=-\sum_{i=1}^{C}y_{i}\log(p_{i})$$

However, at low SNR, modulated signals may contain tag noise, leading to training overfitting and degraded generalization on low SNR signals. Therefore, label smoothing is introduced in this paper. By adjusting the distribution of the real label, the absolutely correct label is softened. The probability of the real category is reduced from 1 to 1−ε, and the remaining *C* − 1 categories are increased from 0 to $\frac{\varepsilon }{C-1}$. The smoothed label distribution *y_smooth_* is

(21) $$y_{i}^{smooth}=\left\{\begin{array}{l} 1-\varepsilon ,i\;\mathrm{is}\;\mathrm{the}\;\mathrm{true}\;\mathrm{category}\\ \frac{\varepsilon }{C-1},\mathrm{others} \end{array}\right.$$

where ε is the smoothing factor (usually $\varepsilon \in \left[0.1,0.5\right]$) and *C* is the total number of modulation classes (e.g., QPSK\16QAM, etc.). Finally, the smoothed labels $y_{smooth}$ are brought into the cross-entropy loss,

(22) $$L_{CE+\mathrm{smooth}}=-\sum_{i=1}^{C}y_{i}^{smooth}\log(p_{i})$$

When expanded, it is divided into two parts,

(23) $$L_{CE+smooth}=-(1-\varepsilon )\log(p_{true})$$

Where the non-true category item $\sum_{i\neq true}\frac{\varepsilon }{C-1}\log(p_{i})$.

Modifying the loss function encourages the model to predict the true class with a probability close to 1−ε, rather than the extreme value of 1, and prevents the model from predicting the other classes with a probability of exactly 0, preserving a certain probability distribution. By adjusting the distribution of the true label, the smoothed model prediction probability is closer to the true confidence, thereby enhancing the model’s generalization capability.

## 3. Experimental Results

### 3.1. Data Set Settings

This paper uses the public data sets of RadioML2016.10a [36] and RadioML2018.01a [37] generated based on GNU Radio 3.7.9 and Python 3.6.4 as the data source for model training and testing. The RadioML 2016.10a data set is generated by simulating the dynamic channel model through GNU Radio. Each sample consists of 128 samples of the IQ two-way signal. Specific data parameters are shown in Table 1.

The RadioML2018.01a data set is large in scale and mixes various types of high-order modulation signal. It also considers key impairments of wireless signals in real environments, and is considered to be the most challenging data set in the field of modulation identification [38], with a total of 24 modulation types. Specific data parameters are shown in Table 2.

In this study, the data set is divided into training, validation, and test subsets in an 8:1:1 proportion, ensuring balanced representation across various modulation types and SNR levels. To eliminate channel gain effects, the symbol waveform signal data is normalized using min–max normalization. This linear transformation maps the original data to the range [0, 1], and the conversion function is specified as follows:

(24) $$\hat{S}=\frac{S_{i}-S_{\min}}{S_{\max}-S_{\min}}$$

The simulation environment of the experiment is shown in Table 3.

### 3.2. Analysis of Experimental Results

**Figure 9:**
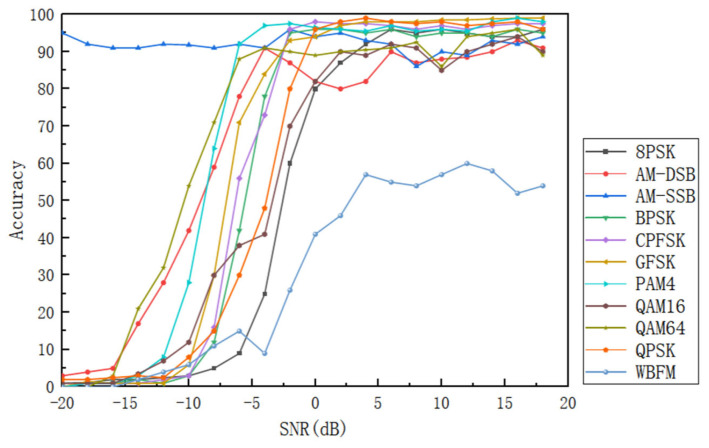
Classification accuracy of different modulation signals at different SNRs.

The network framework in this paper is trained and tested. First, the recognition accuracy is used to evaluate the classification accuracy of the network model. Figure 9 illustrates the modulation classification and recognition accuracy for various modulation signals in the RadioML2016.10a data set at different signal-to-noise ratios.

It can be seen from Figure 9 that, except for AM-SSB and WBFM, the recognition rates of other modulated signals increase with the increase in SNR, and finally tend to be stable with small fluctuations. Among them, the recognition accuracy of CPFSK, GFSK, PAM4, QPSK, BPSK, and 8PSK is close to 100%; the accuracy of AM-SSB is always higher than 90%, and the peak value is more than 95%; while WBFM achieves the highest recognition rate of 61% at 14 dB, and stabilizes at about 50%. It is worth noting that when the signal-to-noise ratio is reduced to −6 dB, the overall recognition accuracy of the model is significantly reduced, which is about 25% lower than that at 0 dB. This limitation is mainly due to the significant improvement of the similarity of high-order modulation signals (such as 16QAM, 64QAM) under low signal-to-noise ratio, and the serious dispersion of their constellations caused by high-frequency noise. Subtle differences in amplitude and phase are masked, making it difficult for the model to distinguish between quadrature amplitude modulation signals of adjacent orders. When the SNR is greater than or equal to 4 dB, the overall average recognition rate is more than 91% and the peak value is 93. 2%, which shows that the model has strong recognition ability for 11 modulation modes.

**Figure 10:**
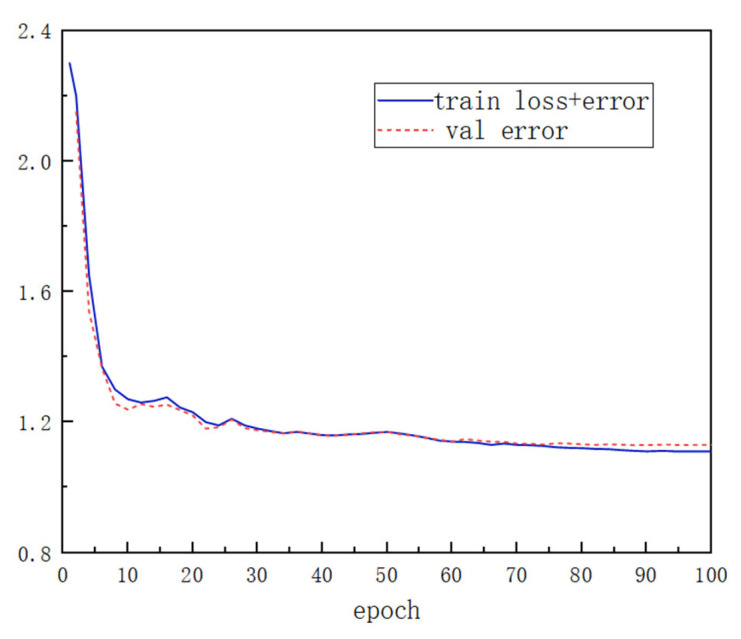
Training and validation loss process.

Figure 10 displays the progression of training loss and validation error across 100 epochs. It can be observed that the training loss gradually decreases and eventually stabilizes. In the training process, we use the early stop strategy to prevent a gradual decline in the performance of the neural network. By stopping the training in advance, the effectiveness of the model can be improved and the overfitting of the network model can be avoided. Specifically, when the generalization error of the validation data set begins to rise, the training will be suspended, thus effectively reducing the overfitting and improving the model’s generalization performance.

#### 3.2.1. Comparative Experiment

**Figure 11:**
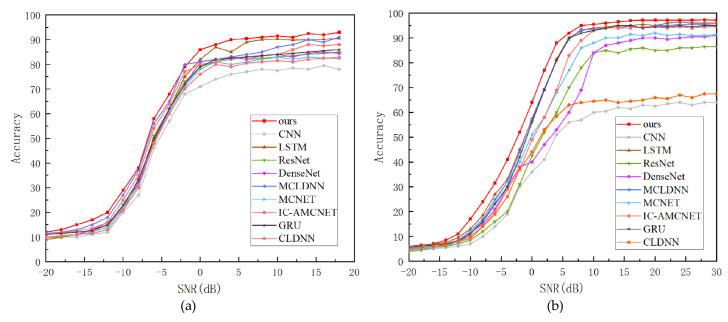
The recognition accuracy of different models. (**a**) RadioML 2016.10a—comparison of recognition accuracy of different models. (**b**) RadioML 2018.01a—comparison of recognition accuracy of different models.

In order to further explore the performance of the model proposed in this paper, the algorithm in this paper is compared with nine typical algorithms, as shown in Figure 11, namely CLDNN [39], GRU [40], IC-AMCNet [41], MCNET [42], MCLDNN [43], DenseNet [44], ResNet [45], LSTM [46], and CNN [47].

Empirical results reveal that all models exhibit monotonically improving classification rates as SNR increases. In high-noise regimes (SNR lower 0 dB), essential signal characteristics are obscured by noise, leading to suboptimal model operation. The performance threshold occurs at an SNR higher than 0 dB, where the extraction of effective information is enhanced, and the recognition effect is gradually stable. The proposed model extracts complementary information through multi-feature input and feature fusion attention mechanism, and outperforms the comparison model at different SNRs. In the RadioML 2016.10a data set, the recognition rates were 86.9% (0 dB) and 93.2% (18 dB); in the RadioML 2018.01a data set, the recognition rates were 63.5% (0 dB), 96.7% (18 dB), and 97.2% (30 dB), respectively. The results show that the model performs well in both high and low signal-to-noise ratios, and has outstanding generalization ability.

For comparison of the overall performance improvement of the proposed method, Table 4 and Table 5 presents the results comparing the model with others across different signal-to-noise ratios (−6 dB, 2 dB, 6 dB, 10 dB, 18 dB, and 30 dB). The algorithm consistently shows improved recognition accuracy across all SNR levels, validating the effectiveness of the deep learning network model constructed in this paper.

#### 3.2.2. Ablation Experiment

**Figure 12:**
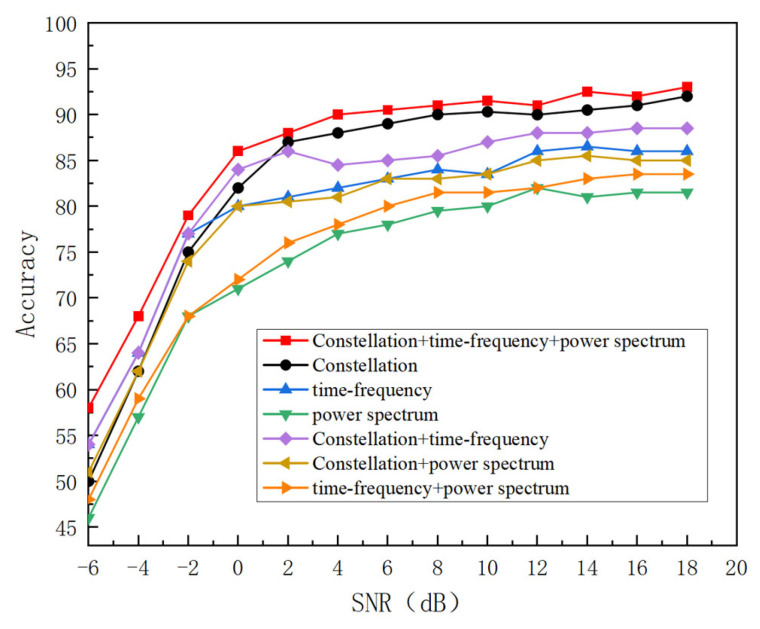
The recognition accuracy of different methods.

To further investigate the effectiveness of the feature fusion module, ablation experiments were performed on the RadioML 2016.10a data set. In order to test the influence of different feature inputs and add an attention mechanism on the recognition effect of the model, experiments are conducted on four schemes, namely, constellation map input, time-frequency map input, power spectrum input, and the multi-feature plus attention mechanism. The comparative results are visualized in Figure 12. As training iterations progress, all four strategies demonstrate progressively higher recognition accuracy across increasing SNR levels. When the signal multi-feature combined with an adaptive attention mechanism is used, through the effective extraction of signal features and the screening of redundant information through the attention mechanism, the recognition effect is the best compared with other models, and the highest recognition rate can reach 93.2%.

In order to further verify the effectiveness of the TDFF module, we fixed other modules on the RadioML2016.10a data set, compared with the non-TDFF module, and directly spliced the multi-dimensional features. The TDFF module fixed the channel weight, set it to 1/3 of the mean value, and only reserved the spatial selection; the TDFF module fixes the spatial weights (set to a mean of 1/64 × 64), keeping only the channel selection and the full TDFF model. The experimental results are shown in Table 6.

The experimental results show that the total gain of dynamic channel and space is 7.2%, and the actual gain of TDFF module is 8.4% through dynamic channel and space co-fusion, which indicates that there is a 1.2% co-gain, and a recognition accuracy of 92.1% is achieved in the range of 0–18 dB SNR, which verifies the effectiveness of multi-dimensional adaptive fusion.

In order to further explore the problem of insufficient channel information interaction in Swin Transformer, we will fix other modules on the RadioML2016.10a data set, and construct networks of different depths with the original self-attention mechanism and CPCA module, respectively. The interaction effect of channel characteristics is quantified by the following indicators: firstly, the Channel Interaction Index (CII), which quantifies the degree of feature interaction between channels. The higher the value, the more fully the channel information is fused. The FDS (Feature Distribution Spread) reflects the distribution diversity of features in the channel dimension. The larger the value is, the richer the distribution is after feature interaction. Classification accuracy was also used, and the experimental results are shown in Table 7.

In the shallow network, the CII and FDS of CPCA are higher than those of Swin Transformer, which indicates that CPCA can aggregate channel features more efficiently; the classification accuracy is improved by 2.7%, and the delay is reduced, which verifies that CPCA can enhance the interaction of basic features and take into account the efficiency in the shallow network. Middle-level features need more complex semantic fusion, and CII and FDS of CPCA are improved more significantly, with a classification gain of 3.5%. It shows that CPCA adapts to the requirements of middle-level feature abstraction, and mines more discriminative semantic information by strengthening channel interaction, while maintaining computational efficiency. Deep network features have complex semantics, CII and FDS of Swin Transformer decrease significantly, while CPCA still maintains high interaction strength, and the classification accuracy increases by 5.2%. It shows that CPCA can break through the bottleneck of deep network channel information interaction, integrate high-order semantic features through precise cross-channel attention allocation, and the delay advantage is sustained, reflecting the superiority of the mechanism.

This study evaluates the effectiveness of the proposed label smoothing loss by comparing it with conventional cross-entropy loss, focal loss, and hinge loss functions. The accuracy rates under different losses of the RML 2016.10a data set are shown in Table 8.

As can be seen from the results of accuracy, the scheme of cross entropy loss combined with label smoothing performs best in this task, providing the highest accuracy. The results demonstrate that label smoothing enhances the model’s modulation recognition performance.

In order to verify the optimal value of the label smoothing factor ε, keep the other parameters of the model unchanged, only adjust ε∈{0.1, 0.2, 0.3, 0.4, 0.5}, adopt 5-fold cross-validation on the training set, and record the accuracy of the training set and the accuracy of the validation set in each validation. The test set is an independently divided data set, and the accuracy of the test set is used as the evaluation criterion of the generalization ability of the model. The experimental results are shown in Table 9.

When ε = 0.3, the accuracy of both the validation set and the test set reaches the peak, and the difference between the accuracy of the training set and the validation set is the smallest, which indicates that the value achieves the optimal balance between suppressing overfitting and retaining label information, and the prediction confidence is closest to the true probability (gap = 0.18); however, when ε > 0.3, the training is obviously insufficient.

#### 3.2.3. Confusion Matrix

**Figure 13:**
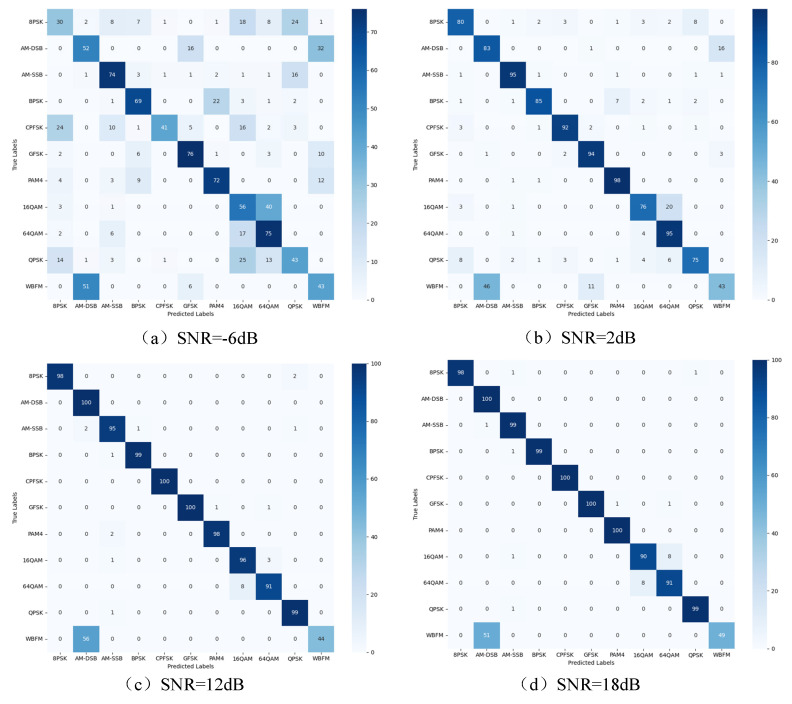
Confusion matrix of RadioML 2016.10a for different SNRs.

**Figure 14:**
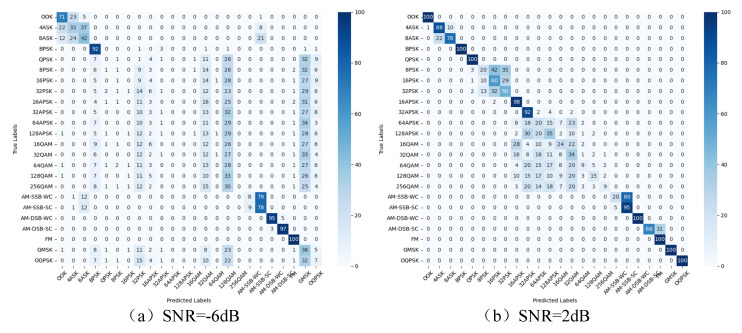

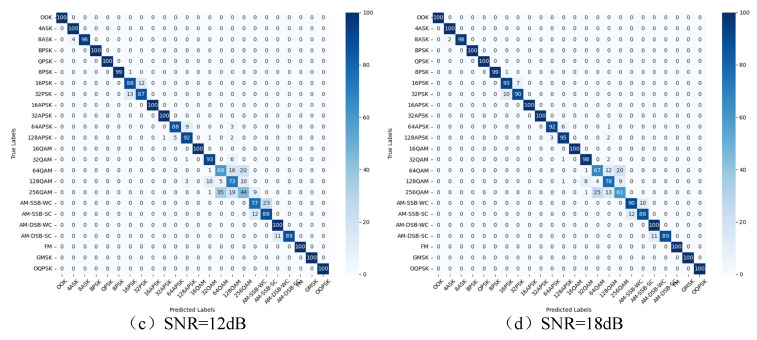
Confusion matrix of RadioML2018.01a for different SNRs.

To further validate the model’s performance under different SNRs in this paper and the effectiveness of identifying various modulation signals, four SNR scenarios are considered in the RadioML2016.10a data set: the confusion matrix of each modulation type under −6 dB, 2 dB, 10 dB, and 18 dB SNRs, as shown in Figure 13 and Figure 14.

The experimental results indicate a significant improvement in model recognition performance as the SNR increases on the RadioML 2016.10a data set. The overall recognition rate is low at −6 dB, but it improves for all modulation types as the SNR rises to 2 dB. but the modulation types such as WBFM, QPSK and 8PSK are still greatly affected by noise interference. With the improvement of SNR, the recognition rate of modulation type is more than 90% at 0 dB. The recognition rate of most modulation types is more than 95% at 10 dB, and the optimal recognition effect is achieved at 18 dB. The model performs well in QAM within-class signal recognition, and effectively improves the classification performance of WBFM and AM-DSB signals, but there are still misjudgments caused by the lack of quiet period features of WBFM signals, the high similarity between 16QAM and 64QAM features in noisy environments, and the 128 point signal length limiting the ability of feature extraction. These factors together restrict the recognition accuracy of the model for specific signal types.

The experiment tests four SNR scenarios of −6 dB, 6 dB, 18 dB and 30 dB in the RadioML2018.01a data set. At 6 dB, the network cannot accurately identify PSK, APSK, QAM, and GMSK signals, mainly due to the serious interference of low SNR and the complex state of high-order signals. At 6 dB, the recognition rate of simple modulation such as OOK and BPSK is 100%, but there is still confusion between APSK, QAM and high-order ASK and PSK signals; when the SNR increases to 10 dB, the recognition rate of most modulations exceeds 85% except for the high-order signals above 64QAM. When the SNR increases to 18 dB, the recognition rate of each signal is close to the peak, but the high-order signals above 64QAM are still confused. The results indicate that higher-order modulation signals are the primary factor affecting recognition accuracy. As the SNR increases, the model’s feature extraction ability improves, leading to a significant boost in recognition accuracy.

## 4. Conclusions

The modulation signal recognition method based on multi-dimensional characteristics fusion proposed in this paper demonstrates clear advantages over traditional single-dimensional feature extraction approaches in dealing with complex environments. Traditional methods usually rely on single-dimensional features, such as constellation diagram or time-frequency diagram, to describe the signal, but these single-dimensional features are often not enough to fully reveal the complexity of the signal, especially in low signal-to-noise ratio environments, the ability of feature extraction is often limited. Therefore, this paper successfully improves the multi-dimensional description ability of the signal by constructing multi-dimensional features including constellation, time-frequency and power spectrum. The multi-dimensional feature representation can not only capture the dynamic changes and time domain characteristics of the signal more comprehensively, but also effectively make up for the shortcomings of a single-dimensional feature in a low signal-to-noise ratio environment. By introducing the TDFF and the CPCA mechanism, the model can make full use of different dimensions of signal features to further improve the recognition accuracy.

Experimental results on two standard datasets show that the proposed model successfully achieves accurate recognition of various modulation types. Under the condition of high SNR, the recognition accuracy of most modulation types is more than 95%. Compared with the existing deep learning recognition methods, the recognition accuracy of this method is improved by 3% to 14%. This significant improvement not only verifies the potential of multi-dimensional feature extraction in improving recognition accuracy, but also proves the superiority of the proposed method in anti-noise performance. Compared with the traditional single-dimensional feature method, the proposed multi-dimensional feature fusion strategy has a stronger generalization ability and can adapt to signal recognition tasks in different noise environments. In short, the intelligent identification method of communication signals proposed in this study provides an efficient technical solution for practical applications, and shows important application value in key areas such as communication signal reconnaissance, spectrum monitoring and system optimization. This method not only significantly improves the accuracy and robustness of signal recognition in complex electromagnetic environments, but also offers both theoretical insights and technical support for research in the field of intelligent radio signal processing.

## Figures and Tables

**Table 1:** Parameters of RadioML 2016.10a data set.

Training Parameters	Parameter Value
Signal Type	Digital modulation: 8PSK, BPSK, CPFSK, GFSK, PAM4, 16QAM, 64QAM, QPSK: analog modulation: AM-DSB, AM-SSB, WBFM
Signal Format/Dimension	In-phase quadrature component (IQ)/2 × 128
Sampling Frequency	200 KHz
Digital Modulation Type Center Frequency	902 MHz
Analog Modulation Type Center Frequency	102 MHz
Total Samples	220,000
Signal-to-Noise Ratio Range	−20:2:18 dB

**Table 2:** RadioML2018.01 data set parameters.

Training Parameters	Parameter Value
Signal Type	32PSK, 16APSK, 32QAM, FM, GMSK, 32APSK, OQPSK, 8ASK, BPSK, 8PSK, AM-SSB-SC, 4ASK, 16PSK, 64APSK, 128QAM, 128APSK, AM-DSB-SC, AM-SSB-WC, 64QAM, QPSK, 256QAM, AM-DSB-WC, OOK, 16QAM
Signal Format/Dimension	In-phase quadrature component (IQ)/2 × 1024
Total Samples	2,555,904
Signal-to-Noise Ratio Range	−20:2:30 dB

**Table 3:** Environment configuration parameters.

Configuration Content	Configuration Parameters
Hardware Configuration	Intel(R) Core(TM) i5-12490F; NVIDIA GeForce RTX 3090
Memory	64 GB
Operating System	Microsoft Windows 10 Professional (64-bit)
Deep Learning Framework	PyTorch 1.9.

**Table 4:** Accuracy of different models in RadioML 2016.10a data set.

Models	Parameters	−6 dB	2 dB	10 dB	18 dB	Highest Accuracy	Average Accuracy	F1 Score	Training Time (s/Epoch)
Ours	18,350,000	58.1	88.3	91.5	93.2	93.2	62.74	62.6	73
CNN	858,123	46.0	74.0	77.5	78.0	79.5	51.88	51.2	17
LSTM	201,099	49.0	87.0	90.3	90.5	90.5	59.17	58.0	11
ResNet	3,098,283	51.0	81.0	83.0	85.0	85.3	56.26	54.9	23
DenseNet	3,282,603	48.0	82.0	84.0	84.5	84.5	56.40	55.0	35
MCLDNN	406,199	56.0	82.0	87.0	91.0	91.1	59.73	58.5	17
MCNET	121,511	48.0	81.0	83.0	83.0	82.6	55.29	53.9	8
IC-AMCNET	1,264,011	54.0	81.0	84.0	88.0	86.5	58.14	56.8	6
GRU	1,51,179	50.0	82.0	84.0	86.0	85.9	56.86	55.5	9
CLDNN	164,433	48.0	80.0	81.5	82.5	82.5	54.55	53.2	8

**Table 5:** Accuracy of different models in RadioML 2018.01a data set.

Models	Parameters	−6 dB	6 dB	18 dB	30 dB	Highest Accuracy	Average Accuracy	F1 Score
Ours	18,350,000	31.5	92.0	96.7	97.2	97.3	64.77	96.8
CNN	858,123	14.0	56.0	63.0	64.0	64.5	40.98	62.0
LSTM	201,099	27.0	90.0	95.5	96.0	96.1	62.04	95.3
ResNet	3,098,283	16.0	70.0	86.0	86.5	87.1	52.17	85.8
DenseNet	3,282,603	21.0	60.0	90.6	91.0	90.6	53.60	89.5
MCLDNN	406,199	25.0	89.5	94.0	95.0	96.1	61.42	94.5
MCNET	121,511	24.0	77.0	91.0	91.5	91.5	57.35	90.2
IC-AMCNET	1,264,011	20.0	83.0	94.0	94.5	94.8	58.59	93.7
GRU	151,179	23.0	90.0	95.0	95.0	95.2	60.86	94.8
CLDNN	164,433	19.0	63.0	65.0	67.5	68.9	43.93	66.1

**Table 6:** The accuracy rate of RadioML 2016.10a data set under different modules.

Configurations	0–18 dB Accuracy
Direct splicing	83.7
Fixed channel weight	86.1
Fixed spatial weight	88.5
Complete TDFF module	92.1

**Table 7:** Performance comparison of modules at different depths.

Depth Type	Module	CII	FDS	Acc
Shallow	Swin Transformer	0.41	1.12	86.4
	CPCA	0.58	1.45	89.1
Middle layer	Swin Transformer	0.37	0.98	89.3
	CPCA	0.63	1.67	92.8
Deep layer	Swin Transformer	0.29	0.81	87.9
	CPCA	0.52	1.39	93.1

**Table 8:** The accuracy rate of RadioML 2016.10a data set under different losses.

Loss Function	0–18 dB Accuracy
Cross Entropy Loss + Label Smoothing Cross	92.63
Entropy Loss	92.39
Focus Loss	91.51
Hinge Loss	91.92

**Table 9:** Accuracy for different values of ε.

ε	Training Set Accuracy	Validation Set Accuracy	Test Set Accuracy	Confidence Gap
0.1	93.8	90.1	89.5	0.29
0.2	92.4	91.3	90.7	0.21
0.3	91.1	91.5	91.2	0.18
0.4	89.7	90.8	90.1	0.23
0.5	87.3	89.2	88.4	0.31

## Data Availability

The original contributions presented in this study are included in the article. Further inquiries can be directed to the author.
